# A critical Response to “How firearm legislation impacts firearm mortality”, A focused look at Canadian and Australian evidence

**DOI:** 10.1016/j.hpopen.2025.100137

**Published:** 2025-03-10

**Authors:** Caillin Langmann

**Affiliations:** Division of Emergency Medicine, Department of Medicine, McMaster University, 1280 Main Street West, Hamilton, ON L8S4L8, Canada

## Abstract

A recent review article in *Health Policy Open*, entitled “How firearm legislation impacts firearm mortality internationally: A scoping review” claims that Australian and Canadian firearms legislation is associated with reductions in homicide and suicide by firearms. Unfortunately, the review overexaggerates the effectiveness of firearms legislation in Australia and Canada, leaves out some important studies, and does not rigorously examine these articles.

Eight Australian studies are referenced that examine the association between gun control legislation, primarily the National Firearms Act (NFA), and firearm homicide. Seven studies find no association between gun control legislation and firearm homicide. Only one study finds a reduction in female homicide but this is contradicted by a study using methods controlling for confounding factors. Four studies examining suicide rates and the association with the NFA find no associated benefit, including the single study that controls for confounders. Two studies find an associated decline in firearm suicide rates with the NFA but there is a decline in non firearms homicide rates at the same time that makes it impossible to know if the decline is associated with the NFA or another variable.

The results of the Canadian studies on legislation and the association with firearms homicide points to no beneficial association when more methodologically sound methods and studies are reviewed. Canadian studies on the association with legislation and suicide by firearm demonstrate a reduction in suicide rates with a substitution for other methods and no overall reduction in suicide rates.

Overall, Australian and Canadian studies to not appear to demonstrate beneficial associations with gun control legislation.

## 1 Introduction

The recent review article in *Health Policy Open*, “How firearm legislation impacts firearm mortality internationally: A scoping review”, Greenberg et al. 2024, appears to overexaggerate the effectiveness of firearms legislation, in particular in Australia and Canada, leaves out some important studies and misclassifies the methods in the Supplementary [1]. Several concerns were noted in multiple areas of the review article, and these will be addressed along with a brief discussion about why more detailed analysis of studies is required when performing a critical review rather than accepting results at face value. Previous reviews of the same studies in the area of firearm legislation and associated effects on suicide and homicide have taken a more comprehensive examination of the validity of methods and results, and demonstrated that gun control is by no means certain to reduce homicide or suicide but rather results in more nuanced findings [2,3].

Australia and Canada have comprehensive gun control legislation that are constructed at the federal level providing a homogenous system of regulations preventing easy bypass of such unlike the United States where gun control is heterogenous due to state level regulations and crossing state lines with firearms is very easy. For this reason, Australia in particular, is often held up as a model for other countries.

## 2 Australia

The review article references Australian studies in its Table 2, under Results section 4.4, and in the Discussion. The text in the table implies that some studies of the National Firearms Act (NFA) in 1996 found no association between the act and homicide and suicide while other studies did. The results section states that “all [thirteen studies] observed a decrease in rates of homicide and suicide except for one” and the fifth paragraph of the Discussion implies a benefit from legislation in particular with regard to homicide. The first issue here is aggregating suicide and homicide by firearm into a single category of firearm mortality; the causes of homicide and suicide differ, and it is theorized that regulations would in some cases have no effect on suicide but a possible effect on homicide, ie: bans of semi-automatic rifles. Thus, it is important to clearly state whether there was an associated benefit regarding suicide or homicide by firearm in association with legislative efforts and not imply that both may benefit.

Next, the statement that “all thirteen studies except for one” demonstrate an associated benefit is completely misleading and does not properly address the nuances of these studies. This is also problematic as that statement only references eleven Australian studies, and as will be discussed below some of these studies do not find a benefit at all. (Please note that the review appears to only reference a total of twelve Australian studies, and one of the studies, #114, referenced in Table 2 appearing to be a misplaced reference to an American study and should refer to #148). Each study referenced will be examined in relative order and referred to by their reference number in the review. The benefits of these Australian studies are the majority use a quasi-experimental design rather than many of the cross-sectional studies comparing states in the United States, with the regulations being an intervention in time series data. These studies therefore tend to be much higher quality of evidence, especially if other associated variables or controls are incorporated into the studies. These studies are summarized in Table 1 of this article.

**Table 1:** Australian Studies of Legislative Association with Firearms Deaths.

Author(s), Year	Method	Law	Findings
Snowdon and Harris 1992	Examination of firearm suicide rates	South Australia Gun Control 1980	No statistical models were constructed Rates of firearm and non-firearm suicide declined
Cantor and Slater 1995	Average firearm suicide rates two years pre and post intervention	Weapons Act of Queensland 1990	Difference between suicide rates pre and post intervention Suicide rates in urban areas declined but no change in rural areas Increase in suicide by other methods
Ozanne-Smith et al. 2004	Time series regression	NFA, Victoria Gun Control	No association between legislation and firearm homicide An associated impact on suicide rates
Chapman et al. 2006	Time series regression	NFA	No association between the NFA and firearm homicide An associated similar decrease in suicide by firearm and non-firearm suicide An associated increase in unintentional firearms deaths
Lee and Suardi 2008	ARIMA	NFA	No association between NFA and suicide or homicide by firearm
Klieve and Barnes 2009	Regression analysis	NFA	No significant change in suicide rates in Queensland A substitution effect between firearms suicide and hanging Does not support NFA having a significant impact on firearm suicide
McPhedran and Baker 2012	Zivot-Andrews	NFA	No breakpoints found, no association between youth suicide by firearm and the NFA
Chapman et al. 2016	Time series regression	NFA	No association between the NFA and firearm homicide An associated decrease in suicide by firearm and non-firearm
Gilmour et al. 2018	Difference in differences Accounts for confounders with quasi experimental design	NFA	No association between NFA and suicide or homicide by firearm in any age group or male or female cohort
McPhedran 2018	ARIMA Zivot-Andrews	NFA	No associated benefit between the NFA and male firearm homicide ARIMA demonstrates associated benefit between the NFA and female homicide but contradictory findings utilizing ZA testing
Ukert et al. 2018	ARIMA	NFA	No statistically significant (p < 0.05) association between the NFA and firearms homicide and suicide

**Table 2:** Canadian Studies of Legislative Association with Firearms Deaths.

Homicide				
Author(s), Year	Method	Law	Findings	
Mauser et al. 1992	Time Series Analysis Socioeconomic factors	C-51	No association with firearm homicide rates Socioeconomic factors associated with homicide rates	
Lester et al. 1994	Mean Averages	C-51	No valid results	
Lester et al. 1996	Mean Averages Pre and Post Trends	C-51	Decline in male firearm homicide rates Switch to other methods for male homicide No association with female homicide	
Carrington 1999	Difference between pre and post trends	C-51	Male homicide by firearm and non-firearm decreased similar rate Females similar decrease Cannot attribute to C-51 since rates of homicide and non-homicide drop same rate	
Leenaars et al. 1999	Time Series Analysis 2 socioeconomic factors	C-51	Decrease in homicide rates associated with C-51	
Leenaars et al. 2001	Mean Averages Time Series Analysis Socioeconomic factors	C-51	C-51 not associated with male and female firearms homicide rates	
Bridges 2004	Mean Averages Linear Regression	C-17	Decline in firearm homicide with similar decline in non-firearm homicide Suggests other factors cause changes	
Blais et al. 2011	Linear Regression Some socioeconomic factors	C-51 C-17 C-68	C-51 barely significant association with decrease in firearm homicide C-17 not associated with decrease in firearm homicide C-68 associated with decrease in firearm homicide Overall homicide rates do not change No legislation associated with homicide by restricted and prohibited firearms Significant issue study does not examine a long enough time pre and post legislation	
Langmann 2012	Time Series Analysis ARIMAJoinpoint Socioeconomic factors	C-51 C-17 C-68	No association with legislation and firearm homicide by type of firearm: long gun, handgun No association with legislation and spousal homicide by firearm Uses multiple methods to test Not a large amount of data pre C-51 Agrees with Bridges 2004, Leenaars et al. 2001, Mauser et al. 1992	
McPhedran et al. 2013	ARIMA Zivot-Andrews Test	C-51 C-17 C-68	No association between legislation and male or female homicide No association between legislation and domestic homicide Agrees with Langmann 2012	
Langmann 2016	Difference-in-differences	C-17 C-68	No association with legislation and male or female homicide by firearm Agrees with McPhedran et al. 2013, Langmann 2012, Bridges 2004, Leenaars et al. 2001, Mauser et al. 1992	
Langmann 2023	Difference-in-differences	C-51 C-17 C-68	No association with legislation and mass homicide incidents or deaths by firearm in male and female cohorts Gradual decline over time of mass homicide incidents and deaths	
**Suicide**				

**Author(s), Year**	**Method**	**Law**	**Findings**	**Substitution**

Rich et al. 1990	Time Series Analysis	C-51	Suicide by firearm decreases Suicide by leaping increases	Yes
Lester et al. 1993	Mean Averages	C-51	No valid results	N/A
Lester et al. 1996	Means Averages Pre and Post Trends	C-51	Male suicide by firearm decreases Male suicide by other method increases Females no effect	Yes
Carrington et al. 1994	Time Series Analysis	C-51	Decline in rates of suicide by firearms Decline in rates of suicide by other methods No Beneficial association	No Both decline
Carrington 1999	Difference between pre and post trends	C-51	Male suicide decrease in trend of firearm suicide Female decrease in trend of firearm and non-firearm suicide	No
Leenaars et al. 1999	Time Series Analysis Some socioeconomic factors	C-51	No effect associated with C-51	N/A No effect
Leenaars et al. 2003	Time series analysis Socioeconomic factors	C-51	Male decline in suicide by firearm with an increase in suicide by other methods Female decline in suicide by firearms but larger decline in other method	Yes in males
Bridges et al. 2004	Mean Averages Linear Regression	C-17	Decrease in suicide by firearm No change in overall suicide rates Suggests switch from firearm to non-firearm	Yes
Caron 2004	Mean rates	C-17	Decrease in suicide by firearm No decrease overall suicide	Yes
Cheung et al. 2005	Time Series Analysis	C-17	Decrease in suicide by firearm in youth 15–19 years Increase in suicide by hanging in youth 15–19 No change in overall rates of suicide	Yes
Caron et al. 2008	Time Series Analysis	C-17	No association with C-17 and suicide rates in males and females In males a switch from firearms to non-firearms suicide	Yes
Gagne et al. 2010	Joinpoint Time Series Analysis Quebec	C-17	Male suicide rates by firearm and non firearm are dropping Poisson regression shows a possible substitution effect	Yes
Langmann 2016	Difference-in-differences	C-17 C-68	No association with legislation and overall suicide rates Switch in males over age 45, and females, from suicide by firearms to hanging No association with prevalence of firearms licence holders and provincial suicide rates suggesting prevalence of a firearm doesn’t predict suicide	Yes

The first study #20, by Lee and Suardi 2008, as correctly indicated by the review did in fact find no association with the NFA and suicide or homicide rates applying autoregressive integrated moving average (ARIMA) analysis to data between 1915 and 2004 [4].

The third study #22 by Gilmour et al. 2018 is the most methodologically valid study to date, examining data between 1978 and 2015 using a difference-in-difference (DiD) model constructing controls to compare each time series models and legislative interventions [5]. The use of controls is important in elevating this study above the others, as it accounts for other potential variables such as socioeconomic factors that may contribute to suicide and homicide rates. In addition, the study also breaks suicide and homicide rates into cohorts by age and sex. Contrary to what the authors report in the review, the Gilmour et al. study finds no statistically observable impact of the NFA on suicide or homicide by firearm.

Study #23, McPhedran 2018, examined male and female firearm homicide over the years 1980 to 2013 using ARIMA and Zivot-Andrews breakpoint tests (ZA) to determine an effect from the NFA [6]. No associated benefits were found between the NFA and male firearm homicide rates. While the results of the ARIMA analysis found a beneficial impact for female homicide rates, the ZA testing was contradictory finding a possible slowing of the declining trend in female homicide rates. This study did not incorporate any confounding variables, and the best conclusion one could make is the effect on female homicide rates of the NFA is equivocal.

Study #24, Snowdon and Harris 1992, studies the suicide rates between the years 1968 to 1989 in Australian provinces and compares them to the rates in South Australia which initiated gun control laws in 1980 [7]. The study is extremely rudimentary by today’s standard, no statistical models were constructed, and little can be gleaned from this, other than the rates of firearms suicide declined in South Australia but the rates of suicide by other methods increased proportionally. Whether this occurred because of legislative efforts, or other reasons could not be concluded.

Similarly, study #148, Cantor and Slater 1995, examined suicide rates before and after the Weapons Act 1990 required Queensland owners of long arms to be licensed by the police [8]. This simple study only examined average suicide rates by firearms two years pre and post the Act, and found a lower rate in urban but no change in rural areas. It is unlikely that only two years of suicide rates are enough to determine a change due to the Act. Interestingly rates of suicide by other methods increased in urban areas. There was no examination for trends pre and post legislation and no controls and therefore the study is rudimentary. Table 2 of the Greenberg et al. review incorrectly references this article but does states that “it is difficult to assess whether the Act was the reason for the decrease”.

Study #25, Ozanne-Smith et al. 2004, examined the effects of gun control regulations in Victoria and the NFA on rates of death by firearm by suicide and homicide over 1979 to 2000 using a time series regression that only examined impacts, not trends and did not control for confounding variables [9]. An associated impact on suicide rates was found but not rates of homicide.

Study #27, Klieve and Barnes 2009, uses suicide data from Queensland between 1990 and 2004 and Australian data from 1968 to 2004 [10]. The data for Queensland is more accurate than general Australian data, and both were analysed for an impact effect using regression analysis. The analysis demonstrated no significant change in suicide rates in Queensland associated with the NFA, and though there was a drop in suicide by firearm rates, there was an increase in suicide by hanging. The authors conclude that their results do not support the NFA being the most significant impact on firearm suicide rate decreases but rather a change in social and cultural attitudes.

Studies #26, #28, and #29 by Chapman et al. 2006, 2016, 2018, are a series of studies examining the association with the NFA and suicide and homicide rates [[11], [12], [13]]. Study #26 and #28 use time series regression analysis to analyze the associated effects of the NFA over the years 1979 to 2003 and 1979 to 2013 respectively. Both studies find no statistically significant association between the NFA and firearms homicide rates. The studies do find an association with the NFA and a decrease in suicide rate by firearm but there is also a decrease in suicide by non-firearm occurring at the same time and thus it is hard to differentiate whether this drop is due to the NFA or other confounders causing an overall drop in suicide rates. Incidentally, study #26 also finds an increase in unintentional firearm deaths associated with the NFA. It is important to note that none of these studies incorporate a control for confounders.

Studies #28 and #29 perform a rudimentary ad hoc analysis of mass shooting events occurring between 1979 and 2013, defining an event as five (5) or more deaths, and the purported impacts of the banning of semi-automatic rifles. It is unclear why five deaths are chosen as a cut-off, as the most commonly accepted definition uses four victims while some use three victims [14]. Thirteen incidents are found pre-NFA and none afterwards. However, the studies do not examine the mass shooting events in detail. Six events were spree shootings of two or three victims that could have been done with any type of firearm. Out of the remaining seven events, five used a firearm type that was not banned by the Australian gun laws, hence if the laws had been in place prior to the NFA these events would have still occurred [15]. Since those studies were published, four other mass shooting events have occurred in Australia. Whether these findings can be applied to other countries is also uncertain, it is interesting to note that the United Kingdom banned semi-automatic rifles in 1986. In the 37 years prior to the ban there were 5 mass shooting events of 4 or more victims. In the 36 years after there have been 4 mass shooting events of 4 or more victims, a statistically insignificant difference. A recent study examining the effects of Canadian legislation, similar in detail to the Australian NFA including bans of a large number of paramilitary-style rifles, on mass homicide by shootings over the years 1974 to 2020 also demonstrates no changes in mass homicide rates [16].

It is interesting the authors of this review include reference #21, Ukert et al. 2018, that performs sensitivity testing on Chapman et al. 2016, and finds statistically significant results occurring outside of the intervention periods, and no statistically significant results (p < 0.05) using ARIMA analysis [17]. This suggests that any results Chapman found were due to the use of an empirical model that could not identify the true effects of firearms laws.

Finally, even though the review includes reference #30, Results section 4.4 does not mention the results of McPhedran and Baker, 2012, that performed ZA tests on youth suicide rates over the years 1907 to 2007 [18]. No breakpoints are found associated with the NFA, demonstrating no beneficial association.

The final conclusion from the summation of these Australian articles does not point to a link between the NFA and a reduction in homicide rates by firearm. All but one study, McPhedran 2018 that finds a weak link with female homicide, does not find an association between Australian firearms regulations and homicide rates by firearm. Additionally, the significant flaw in these studies are that none control for confounding variables except for Gilmour et al. 2018, and that study does not find an association between female homicide rates by firearm and the NFA [5].

Four studies examining suicide rates and the NFA find no associated benefit, including the single study that utilizes a control, Gilmour et al. 2018 [4,5,10,17]. One study finds an associated decline in suicide rates when earlier laws are implemented in Victoria compared to the rest of Australia [9]. Two studies find an associated decline in firearm suicide rates with the NFA but there is an associated decline in non firearms homicide rates at the same time that makes it impossible to know if the decline is associated with the NFA or another variable [11,12]. The best determination that can be made from this is that the majority of studies, including the best methodological study available, find no associated benefit.

Therefore, the actual findings of the Australian studies differ markedly from the implication made by the Greenberg et al. 2024 review article in *Health Policy Open*, that point to a benefit associated with strict Australian firearms regulations and firearms mortality in section 4.4 of results and the Discussion. Rather, the results of the preponderance of Australian evidence point to no beneficial association between these regulations and homicide and suicide rates.

## 3 Canada

As described in the review, Canada passed legislation regarding firearms in 1977, 1991, and 1995 referenced as Bill C-51, Bill C-17, and Bill C-68 respectively. Canadian studies of the legislation are more nuanced and need to be carefully examined in terms of the quality of the methodology before conclusions are made. In particular, earlier studies suffer from a significant methodological flaw that makes most of their conclusions invalid. This has been mentioned and reviewed in two previous reviews, Langmann, 2021 and Bennet et al. 2022, and will be discussed below [2,3]. The resulting conclusions from these two review articles regarding suicide by firearm and association with legislation are mixed, involves substitution of other methods, and may not result in a decrease in overall suicide rates. Bennet et al. 2022 found eleven relevent studies, nine reported a beneficial association between suicide and firearms legislation, but seven found method substitution where a switch to other methods of suicide was observed [2]. Their conclusion was that although rates of firearm suicide were reduced method substitution likely occurred as overall rates of suicide did not change. Langmann, 2021, found thirteen relevant studies, and concluded that while an associated decline in firearm suicide was observed, overall suicide rates remained unaffected due to method substitution [3]. The nature of these results is not well explained in the Greenberg et al. 2024 review either in Table 2 or section 4.7 where the simple statement “noted a decrease” does not adequately describe the possibility of method substitution or limited effects of legislation on overall suicide rates. Suicide by firearm is well covered in Langmann, 2021, and will not be discussed in depth here. The Canadian studies are summarized in Table 2 of this article.

Many early Canadian studies used a method which will be called “Mean Averages” for the purposes of this article. This type of methodology, possibly the best that could be done in the past with computer power and available statistical methods, would not be acceptable today. Basically, this method reports the mean homicide or suicide rate prior to an intervention and afterwards. However, the method cannot explain the trend or rate of change over time prior to or after an intervention. A study could therefore find a situation where a difference in mean rate occurs before and after an intervention despite the trend in homicide falling at the same rate at both times. The studies interpret this as being a beneficial effect due to legislation, but the reality is that this cannot reflect any benefit as the rate of decline is similar before and after an intervention.

A simple example can make this clear. Imagine a situation where there are eight people in a room, and every hour one person leaves. Three hours later, three people have left and there are five people remaining. At this point there is an intervention where someone instructs everyone to leave the room. After the intervention, people continue leaving at a rate of one person an hour. There is no rate change. Three hours later three more people leave. The mean number of people prior to the intervention is seven people, and afterwards the mean is four people.

The mean averaging method would interpret the above situation as a change in the rates of people in the room due to the intervention (someone telling everyone to get out). But the reality is that people were leaving at the rate of one person per hour both before and after the intervention. No actual rate change. This illustrates how studies that report mean averages make the mistake of interpreting an effect in homicide or suicide rates where there cannot be an observed effect. Any results from studies using this method should therefore be discarded as invalid.

An example of the use of mean averages is Lester et al., 1994, where the effects of C-51 on homicide is examined [19]. This study reports a drop of a mean homicide rate from 0.96 per 100,000 per year prior to C-51 to a rate of 0.82 per 100,000 after 1977. Unfortunately, as discussed above nothing can be inferred from this as there is no examination of trends pre and post C-51. However, if one is simply reading abstracts and not examining methodology one would mistakenly infer a positive association from this study, which Greenberg et al. do in Table 2 and section 4.7.

The series of studies by Leenaars and Lester demonstrate gradual improvement in methodology with each additional examination of C-51 and homicide rates. Leenaars and Lester, 1996, examines homicide rates with a disaggregation of male and female homicide rates using a similar mean averages method, but does include trend analysis but no statistical attempt to differentiate trend significance and unfortunately no conclusions can be made [20]. More interesting, Leenaars and Lester, 2001, using linear regression on homicide rates from 1969 to 1985 and inclusion of socioeconomic factors such as unemployment, find that there is no statistically significant association of C-51 with male and female homicide rates [21]. Unfortunately, Leenaars and Lester, 2001, continues the use of mean averages as well, which leads to uninterpretable results for the purposes of determining if there was an associated benefit between legislation and homicide rates.

One of the first studies to use modern statistical methods to examine C-51 and homicide rates over the years 1968 to 1988 is Mauser and Holmes, 1992, which uses time series analysis as well as the inclusion of socioeconomic variables [22]. This study finds no association between C-51 and homicide rates and does not suffer from some of the methodological issues mentioned previously. This study is not mentioned by the Greenberg et al. review and it is unclear why as the Bennet et al., 2022 review using the NewCastle-Ottawa Score for risk of bias rated it as high quality [2].

Carrington, 1999, critical of the Leenaars and Lester mean averaging method, examine the homicide trends pre and post C-51 over the years 1969 to 1985 and find similar drops in homicide rates by firearm and non-firearm for males and females which demonstrates no associated benefit that can be attributed to C-51 [23]. This study does not include socioeconomic variables.

Bridges 2004 examines the association between C-17 and homicide rates over seven years pre and post 1991 [24]. Unfortunately, it also uses the mean averages methods. However, linear regression on homicide rates with C-17 as the intervention does find a decline in firearm homicide rates, but also find a similar decline in non-firearm homicide rates, meaning that some other factor(s) than C-17 could be involved in overall homicide rate changes at that time. As well this study also does not include other independent variables.

Blais et al., 2011, examine the association between Bill C-51, C-17, and C-68 and firearm homicide rates over 1974 to 2004 [25]. Linear regression is used and some possible independent variables are included in the analysis. An association with Bill C-51 and C-68 and a decline in firearm homicide rates are found, but no association with Bill C-17, and overall homicide rates do not change. The study suffers from several issues, it uses linear regression instead of the more generally accepted Poisson or negative binomial regression used for count data where negative values are impossible (cannot have a negative number of deaths a year) and thus subject to error. Moreover, many independent variables end up being left out of the analysis due to issues with multicollinearity. Another issue is the application of results, the study only analyses a period of six years after the implementation of Bill C-68 which provides only a small number of years to establish a post-intervention trend and this would be subject to outliers and underlying cyclical effects. It is notable that the authors found no benefits from regulations on the control of military style firearms such as semi-automatic and automatic rifles. They claim that there may be a benefit on the control of nonmilitary style long guns (rifles) due to mandatory registration of these firearms, however registration was not implemented until 2003 thus making that association impossible [26]. Finally, Blaise et al, 2011, only examines Bill C-51 using 3 years prior to the legislation to establish a trend, that is once again prone to error. Mauser and Holmes, 1992, use a much longer pre intervention time frame, starting in 1968 as well as multiple independent variables to produce what is probably a more accurate analysis and find no benefit. The best that can be derived from the Blais et al, 2011, study is that there may be an associated benefit between some regulations such as stricter criminal sentences, improved background checks, and possibly licensing, and homicide by firearms after C-68 but whether this is sustained over a longer period is unlikely considering the results from more recent and more methodologically rigorous studies.

Langmann, 2012, uses binomial time series regression analysis, ARIMA, as well as joinpoint to examine the association between Bill C-51, C-17, and C-68 and firearm homicide over the years 1974 to 2008 [27]. This study disaggregates homicide by type of firearm (long gun, handgun, as well as male, female, and spousal homicide rates. It also includes multiple socioeconomic independent variables into the models. No associations between legislation and homicide are found in this study, and instead socioeconomic factors such as unemployment and population age are associated with changes in homicide rates.

Langmann, 2020, uses a difference in differences model (DiD) to examine the association between Bill C-17 and Bill C-68 and firearm homicide rates over the years 1981 to 2016 [26]. The DiD model essentially constructs a quasi-experimental study using non firearm homicide as a control for independent variables. This study also found no association between legislation and firearm homicide in males and females over several age cohorts.

Finally Langmann, 2023, examined mass homicide rates over 1974 to 2020 to determine if improved background checks, licensing, psychological questionnaires, and prohibition of paramilitary rifles and magazine capacities were associated with changes in mass homicide rates [16]. No association between any of these regulations was found with rates of mass homicide in males or females.

It is interesting to point out that Langmann, 2012, and Langmann, 2020, were not included in the Greenberg et al. review, even though these studies received the highest quality NewCastle-Ottawa Scores in the Bennet et al., 2022, review [2].

The final study to be examined is McPhedran and Mauser, 2013, which uses ARIMA and Zivot-Andrews (ZA) testing to examine for associations between Bill C-51, C-17, and C-68 and homicide of females by firearms over the years 1974 to 2009 [28]. The ARIMA analysis finds no association between legislation and homicide of females by firearms. The ZA analysis finds a breakpoint at around 1982 which may suggest an association with the implementation of Bill C-51 a few years earlier. However, independent variables are not included in this analysis and it is unclear if this result is valid.

Overall, the results of the Canadian studies on legislation and the association with firearms homicide points to no beneficial association when more methodologically sound methods and studies are reviewed. This significantly contrasts with the statements made in the Greenberg et al. review, section 4.7 that “all studies noted a decrease [in homicide] apart from one”.

## 4 Discussion

Studies from Australia and Canada regarding the associations of legislative efforts on mitigating homicide and suicide appear to agree in that there appears to be no statistically measurable effect on homicide, and limited effects on suicide with many studies finding method substitution. While some studies may make broad claims, it is important for any critical reviewer to delve into the methodology and results to determine if there is any validity in the findings as has been done in this article. It seems that Greenberg et al. perform a very limited examination of the evidence, have incorrectly interpreted some studies, have left out others, and therefore either neglect to describe important findings and thus reach deficient conclusions. Leaving out three high quality studies, Mauser and Holmes, 1992, Langmann, 2012, and Langmann, 2020, that demonstrate no beneficial association between firearms legislation and mortality rates is a critical mistake in the Greenberg et al. review. In their Discussion, the claim that Canadian “Bills C-51, 17, and 68 […] showed a trend toward reduced mortality” is both incorrect and misleading. As described in this paper, there appears to be no associated effect on homicide and there does not appear to be a reduction in overall suicide rates as well implying no apparent overall associated benefit between firearm legislation and mortality. Statements about the effectiveness of Australian legislation within the text and discussion are also similarly misleading. Only one out of eleven studies examined found a tenuous link between Australian firearms legislation and homicide rates, four studies show no benefit with regard to suicide rates while two find similar declines in suicide rates by firearms and other methods making it not possible to determine if this was due to legislation or some other factor. This is hardly supportive of the assertion that firearms control legislation corresponds with changes in mortality rates.

Gun control can be extraordinarily expensive, the Canadian long gun registry implemented under C-68, may have cost as much as $2 billion Canadian dollars, was found to be largely ineffectual and eventually cancelled by the Canadian government [3,26,27,29]. The current proposed Canadian gun “buyback” may also cost a similar amount [30]. It is therefore important to take a close look at higher quality quasi experimental studies and make accurate assessments of the evidence before pursuing public policy. What seems theoretically beneficial may be practically ineffective. Programs that target criminal activity and youth diversion appear to have some supportive beneficial evidence, directing funding and targeting these areas may have more significant impacts on homicide [31,32]. For instance, a recent study by Public Safety Canada demonstrated significant reductions in behavior related problems such as violent and non-violent offending behaviour [33].

While the Australian evidence does not demonstrate a clear association with legislation and reduction in suicide, the higher quality Canadian studies do show an association but multiple studies demonstrate substitution into other methods and no overall reduction in suicide rates. While this is may be due to legislation reducing availability or prevalence of firearms, it could also be associated with changes in preferences and social stigma [3]. Clearly a better strategy is needed to reduce overall suicide rates and gun control in and of itself may have no measured benefit compared to screening and interventions such as dialectical behavioral therapy [34]. In Canada significant barriers and wait lists delay mental health care, directing funding to these areas may have a higher degree of return than additional costly gun control programs [35].

## CRediT authorship contribution statement

**Caillin Langmann:** Writing – review & editing, Writing – original draft, Methodology, Investigation, Formal analysis, Data curation, Conceptualization.

## Declaration of competing interest

The authors declare that they have no known competing financial interests or personal relationships that could have appeared to influence the work reported in this paper.
